# Characterization of Odors of Wood by Gas Chromatography-Olfactometry with Removal of Extractives as Attempt to Control Indoor Air Quality

**DOI:** 10.3390/molecules23010203

**Published:** 2018-01-18

**Authors:** Ru Liu, Chen Wang, Anmin Huang, Bin Lv

**Affiliations:** Research Institute of Wood Industry, Chinese Academy of Forestry, Beijing 100091, China; liuru@criwi.org.cn (R.L.); wonderfulmorning@163.com (C.W.); zj3@caf.ac.cn (B.L.)

**Keywords:** wood, odor, volatile organic compounds, gas chromatography-olfactometry

## Abstract

Indoor air quality problems are usually revealed by occupants’ complaints. In this study, the odors of two types of hardwood species, namely, Cathy poplar (*Populus cathayana* Rehd.) and rubberwood (*Hevea brasiliensis*) were selected and extracted with ethanol-toluene for removal of extractives in an attempt to eliminate the odors. The odorous components of neat and extracted woods were identified by gas chromatography-mass spectrometry/olfactometry (GC-MS/O). The results showed that about 33 kinds of key volatile compounds (peak area above 0.2%) were detected from the GC-MS, and about 40 kinds of odorants were identified from GC-O. The components were concentrated between 15 and 33 min in GC-O, which was different from the concentration time in GC-MS. Lots of the odors identified from GC-O were unpleasant to humans, and variously described as stinky, burnt, leather, bug, herb, etc. These odors may originate from the thermos-oxidation of wood components. After extraction, the amounts and intensities of some odorants decreased, while some remained. However, the extraction process resulted in a benzene residue and led to increased benzene odor.

## 1. Introduction

Odor has a direct effect on human behaviors and can significantly affect the quality of life [1]. It certainly plays an important role in human attractions, memories, and emotions, and can be described as pleasant, unpleasant, or indifferent [2]. Therefore, odor is one of many important factors for indoor air quality. Generally, the indoor air quality is strongly affected by volatile compounds emitted from materials such as furniture, carpet, textile, plant and humans [3,4,5]. Among these, wood has been widely used indoors for a long time. Although the odor of wood is often described as pleasant with positive associations, some people are hypersensitive to certain species like padauk (*Pterocarpus indicus* Willd.). Besides, people are concerned with several issues regarding odors, such as the relationship between unpleasant odors and health problems [6]. The odor of wood originates from the volatile organic compounds (VOCs) which are highly relevant to the extractives [7].

Wood extractives are rich in volatiles, typically with several hundred different constituent VOCs in individual species [8,9,10]. Although many of the VOCs are not odorous in nature, a broad overview of the volatiles hitherto detected in diverse woods offers insights into prospective main odor-active candidates that contribute to wood odor [11]. Therefore, numerous studies have been carried out for identification of the specific class of extractives [12,13,14,15,16]. However, not all VOCs contribute to odor because it depends on whether the concentration of the VOC is higher than the human threshold odor concentration [2]. Hitherto, there are few reports on the odor of natural woods. One particular study on the odor-active substances of wood was carried out by Culleré et al. [17] who investigated the odor of acacia, chestnut, cherry, ash and oak chips by gas chromatography-olfactometry (GC-O). Different odorants were detected, including phenolic compounds, as well as compounds arising from the degradation of wood carbohydrates and lipids. Similar studies were performed based on GC-O on toasted and non-toasted oak woods by Díaz-Maroto et al. [18], Poplar, Pine, and Basswood by Wang et al. [19], and cedar by Schreiner et al. [20].

GC-O consists of gas chromatography-mass spectrometry (GC-MS) coupling with an olfactometric detection. It is essential to identify compounds with odor because they are usually a minor set of eluting compounds, which depends on the human nose and shows high sensitivity and reproducibility [21]. It has been widely used in many areas ranging from daily necessities to chemical products [22,23,24,25,26,27]. Therefore, it can be an optimal instrument for odor testing. With the developing of costumed furniture, the wood products are heavily used indoors, and the odor annoyance arises due to the woods. Cathy poplar (*Populus cathayana* Rehd.) is a kind of fast growing tree and widely used for costumed furniture in China. Rubberwood (*Hevea brasiliensis*) is also been used for solid wood flooring and furniture while it contains lots of gum with strong smells. Both of the woods can affect the indoor air quality. Therefore, in this study, the two types of woods were selected for odor identification. Considering the high connection between extractives and odor, the woods were extracted with ethanol-toluene as an attempt to eliminate odor. The aim of this study was to investigate the odors of the two kinds of wood and whether the extraction can be a good method to eliminate the odor, which is important for the controlling of indoor air quality. The odorous components of neat and extracted woods were identified by GC-MS/O. Besides, the chemical components of the woods are analyzed.

## 2. Results and Discussion

### 2.1. Chemical Components

The results of the chemical components of the wood samples are listed in Table 1. According to the related standards, the deviations in the parentheses should not be larger than 0.04%. The analysis can be helpful for better understanding the contents of chemical components as well as extractives in the woods. It can be seen that the extractives of RW were higher than that of CP. The α-cellulose contents of the two woods were almost the same, while the content of holocelluloses of RW was higher than that of CP. By calculating the content differences between holocelluloses and α-cellulose, a rough estimate content value of hemicelluloses can be obtained [28]. The results indicated the RW owned the higher hemicelluloses content than CP. As for the lignin contents, RW was higher.

### 2.2. Identification of VOCs

**Figure 1:**
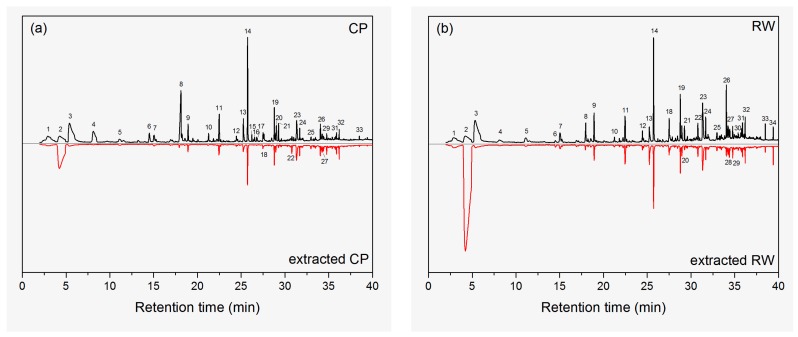
GC-MS chromatograms of neat and extracted wood samples. Peak numbers are listed in Table 2.

As regards the origin of the compounds detected by GC-O, it must be taken into account that only some of the VOCs extracted from the woods are originally present in this material in significant amounts. Therefore, it was very important to know the key VOCs extracted from the woods. Figure 1 shows the GC-MS chromatograms of neat and extracted wood samples. The key compounds with peak area above 0.2% are detected from the chromatograms and the results are listed in Table 2. In general, these compounds can be classified into alkanes, aldehydes, alcohols, aromatics, ketones, carboxylic acids, esters, and miscellaneous (silane derivatives). About 33 kinds of key VOCs were identified in both CP and RW. However, the types and contents of the VOCs were varied like the content of 2,4-di-*tert*-butylphenol (peak 26) was much higher in RW than that in CP. CP and RW had some common VOCs, such as ethanol (peak 1), benzene (peak 2), acetic acid (peak 3), and so on. Some are unique for certain wood. For example, hexyltrimethoxysilane (peak 15), 4-oxononanal (peak 16), and (*E*)-2-decenal (peak 17) were not identified in RW, while tetradecanal (peak 28), 2-methyl-hetadecane (peak 30), and dibutyl phthalate (peak 34) were not identified in CP. Among these compounds, the tetradecane (peak 14) showed the largest intensity, but the percentages were not higher than acetic acid (peak 3). Hexanoic acid (peak 8), tridecane (peak 11), pentadecane (peak 19), 1-dodecanol (peak 23) also had relative high intensities compared with other compounds.

The ethanol-benzene extraction process resulted in significant reduction in the contents of VOCs. For example, the percentages of acetic acid (peak 3) were 14.8% and 16.3% in neat CP and RW, respectively. After extraction, the values both decreased to 1%. Besides, the contents of some compounds in CP like hexanal (peak 4), furfural (peak 5), 1,3-dichlorobenzene (peak 7), pentanoic acid (peak 6), heptanoic acid (peak 10), octanoic acid (peak 12), hexyltrimethoxysilane (peak 15), 4-oxononanal (peak 16), (*E*)-2-decenal (peak 17), *n*-octyltriethoxysilane (peak 21), and 1,2-benzene-dicarboxylic acid bis(2-methylpropyl) ester (peak 33) significantly decreased to lowest detecting limits. Therefore, there were only 20 kinds of key compounds identified after extraction. Similar results were found in RW, suggesting the removal of these compounds. Considering the classification, the contents of aldehydes, carboxylic acids, and miscellaneous considerably decreased. This should be explained by their non-polar characters and solubility in ethanol-benzene.

Pelaez-Samaniego et al. [29] had extracted wood fiber with hot water, and also mentioned the content of acetic acid and aromatics rapidly decreased. The amounts of alkanes, alcohols, ketones, and esters almost remained, which indicated the extraction method has little effect on the removal of these compounds. Aromatics of 1,3-dichloro-benzene (peak 7) also showed considerably reduced contents after extraction. It should be noted that the contents of pentadecane (peak 19), (*E*)-6,10-dimethyl-5,9-undecadien-2-one (peak 22), hexadecane (peak 24), 2,6,10-trimethylpentadecane (peak 25), 2,6,10,14-tetramethyl-pentadecane (peak 27), cedrol (peak 29), (4-octyldodecyl)-cyclopentane (peak 31), benzoic acid 2-ethylhexyl ester (peak 32) increased in CP after extraction, which might account for the removal of other compounds while the contents of these components were remained. Another remarkably increased intensity after extraction is found for benzene (peak 2), which should be associated with the solvent residue.

### 2.3. Characterization of Odors

**Figure 2:**
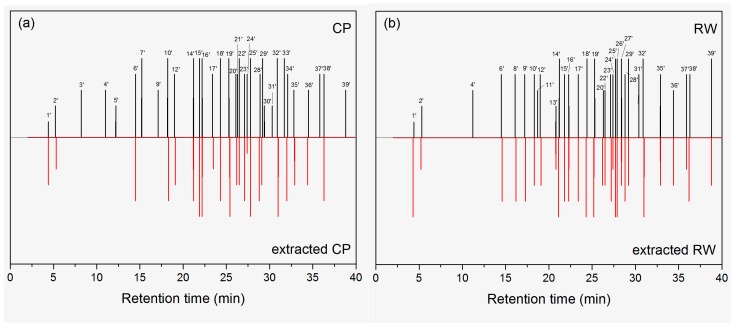
Odor images of neat and extracted wood samples tested by GC-O. Peak numbers are listed in Table 3.

The odor images of neat and extracted wood samples tested by GC-O are shown in Figure 2. About 40 kinds of odors were identified. The time was concentrated from 15 to 36 min, which was different with the concentration time of VOCs in GC-MS, indicating that some VOCs did not contribute to the odors. Table 3 lists the odorants and odor descriptors of neat and extracted wood samples. These odors are described according to judgments of the panelists and then amended on the basis of the literatures [22,23,24,25,26,27]. From Table 2, it is clear that most odors are unpleasant. These odorants can be classified into alkanes, aldehydes, alcohols, aromatics, carboxylic acids, esters, and miscellaneous (silane derivatives). However, these compounds were not corresponding to the key VOCs. For example, some high concentration of alkanes like tridecane (peak 11), tetradecane (peak 14), pentadecane (peak 19), and hexadecane (peak 24) had small smells. Among the alkanes only 2,6,10-trimethylpentadecane (peak 27) offered an odor, which was described as gasoline and bronze- like. Félix et al. [30] investigated the odor of wood-plastic composites and also found little influence of alkanes on the odor of the composites. Some compounds lower than 0.2% caused significantly strong smells for both CP and RW, such as 2-nonenal (peak 15’), 1-methoxy-4-(2-propenyl)-benzene (peak 16’), 5,5,8-trimethyl-3,6,7-nonatrien-2-one (peak 17’), (*E*,*E*)-2,4-decadienal (peak 25’) and 8-methyl-1-undecene (peak 32’). Between the retention times of 20 to 36 min, lots of odorants are emitted. However, these compounds are unpleasant to humans, except for hexadecanoic acid (21’) at 26.3 min in CP and nonanoic acid (peak 24’) at 27.4 min. These unpleasant compounds are usually described as stinky, burnt, leather, bug, herb, etc. Ezquerro et al. [31] mentioned that the unacceptable odor in packaging materials, such as the hexanoic acid is related to the thermo-oxidative degradation of cellulose. Therefore, the extraction method cannot remove it. At the later stage of testing from 37 to 40 min, some perfumed smells existed and the odors are described as milk, fruit, cake, and grass.

For neat CP, 34 kinds of odors were identified, where seven kinds were unique. Hexanal (bitter, green) was identified in both CP and RW. However, the percentage was only 1.2% in RW, which was 1/5 to that in CP. Therefore, the odor intensity was 0 in RW. Another six unique odors were not identified in GC-MS, while they all had strong smells. They were 2-furancarboxaldehyde (special, grain), octanal (fruit, soap), hexadecanoic acid (fruit, sour), 2-methylpropanoic acid, 3-hydroxy-2,4,4-trimethylpentyl ester (sour, bitter, herb), 1-tetradecanol (dirt, bronze), and 1-pentadecanol (bronze). The very strong odors are 12 kinds: octanal (fruit, soap), hexanoic acid (cheese, fishy), heptanoic acid (stinky, leather, bug), 2-nonenal (stinky, fat, iris), 1-methoxy-4-(2-propenyl)-benzene (bitter, herb, burnt), octanoic acid (stinky, fat, bug), (*Z*)-2-decenal (stinky, fat, herb), 4-oxononanal (herb), (*E*,*E*)-2,4-decadienal (animal, burnt), *n*-octyltriethoxysilane (burnt, bug), 8-methyl-1-undecene (paint, dirt), and 1-tetradecanol (dirt, bronze). These odors are all unpleasant. For neat RW, 32 kinds of odors were identified, where five kinds were unique. These compounds were 5-methyl-2-furancarboxaldehyde (chocolate, coconut), 2-octenal (bitter), α-methyl-α-2,5,7-octatrienylbenzenemethanol (bitter, nut), 3,7-dimethyl-2,6-octadien-1-ol, acetate (stinky, bug), and 2-undecenal (citrus, fishy, herb), which were trace in the amount of VOCs. The very strong odors of RW were heptanoic acid (stinky, leather, bug), octanoic acid (stinky, fat, bug), (*Z*)-2-decenal (stinky, fat, herb), (*E*,*E*)-2,4-decadienal (animal, burnt), 3,7-dimethyl-2,6-octadien-1-ol, acetate (stinky, bug), 2-undecenal (citrus, fishy, herb), *n*-octyl-triethoxysilane (burnt, bug), 8-methyl-1-undecene (paint, dirt), and 1,2-benzenedicarboxylic acid, bis(2-methylpropyl) ester (cake, grass), where 8/9 were unpleasant. By comparing the odors between CP and RW, the dominant odorants (intensity above 4) were 16 kinds: pentanoic acid (cheese, paint), hexanoic acid (stinky, leather, bug), nonanal (flower, fat), heptanoic acid (stinky, leather, bug), 2-nonenal (stinky, fat, iris), 1-methoxy-4-(2-propenyl)-benzene (bitter, herb, burnt), 5,5,8-trimethyl-3,6,7-nonatrien-2-one (stinky, chemical, bug), octanoic acid (stinky, fat, bug), (*Z*)-2-decenal (stinky, fat, herb), (*E*)-2-decenal (kerosene, herb), nonanoic acid (milk), (*E*,*E*)-2,4-decadienal (animal, burnt), dodecanal (mint, pungent), *n*-octyltriethoxysilane (burnt, bug), 8-methyl-1-undecene (paint, dirt), octadecanal (bronze, grease), and benzoic acid, 2-ethylhexyl ester (milk, fruit).

After ethanol-benzene extraction, both the amounts and intensities of odors decreased in both CP and RW, suggesting the extraction process had removed some kinds of odorants, such as hexanoic acid (cheese, fishy), nonanal (flower, fat), *n*-octyltriethoxysilane (burnt, bug), decanoic acid (herb), octadecanal (bronze, grease), and 1,2-benzenedicarboxylic acid, bis(2-methylpropyl) ester (cake, grass) and so on. However, some kinds odors remained their intensities, such as acetic acid (irritant, sour), pentanoic acid (cheese, paint), 2-nonenal (stinky, fat, iris), 1-methoxy-4-(2-propenyl)-benzene (bitter, herb, burnt), (*Z*)-2-decenal (stinky, fat, herb), (*E*)-2-decenal (kerosene, herb), (*E*,*E*)-2,4-decadienal (animal, burnt), dodecanal (mint, pungent), 8-methyl-1-undecene (paint, dirt), cedrol (fat, wood), benzoic acid, 2-ethylhexyl ester (milk, fruit) and so on though the amount percentages was reduced in GC-MS. In addition, consistent with the results of GC-MS, the residue of benzene caused increase of odor, which was described as gasoline and solvent.

As mentioned above, the uncomfortable odors originated from the thermos-oxidation. Maybe the temperature had a tremendous influence on these odorants. Further study can focus on the effects of temperature on the odor release or try to find another effective odor elimination process.

## 3. Materials and Methods

### 3.1. Materials

**Figure 3:**
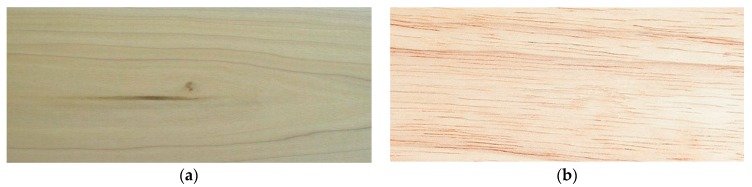
Images of tangential sections of the two kinds of wood. (**a**) CP; (**b**) RW.

The two kinds of wood were harvested from forests and then bucked into lumbers. The lumbers were air dried for at least 3 months to eliminate excessive water. Both the kinds of wood were provided by at least three providers from a single origin and analyzed to ensure their uniformity. The average growth ring widths, densities and origins of place are presented in Table 4. The sapwood was chosen without bark and visible defects such as knots, decay, and so on. Figure 3 shows tangential sections of the two kinds of wood. The wood samples were ground into fibers of 40–60 mesh consistent with the particle sizes about 50 μm.

### 3.2. Analyisis of Chemical Components

The wood fiber was extracted in a Soxhlet extractor with a 1:2 mixture of ethanol and toluene (*v*/*v*) for 6 h, followed by a second extraction with ethanol for 4 h to remove extractives. The extracted wood fiber was dried in an oven at 103 ± 2 °C to reach a constant weight. The content of extractives was calculated. The chemical components of the natural fibers for holocelluloses, α-cellulose, and lignin contents were performed according to chlorite method, TAPPI 203 cm-09, and TAPPI T 222 om-11, respectively [32].

### 3.3. Odor Characterization

The odor characterization experiment was tested by a GC-MS (QP-2010, Shimadzu, Shimane, Japan) combined with an olfactory port (OP 275, GL Sciences, Shimane, Japan) connected by a flow splitter to the column exit. Separation was achieved using a DB-WAX column (30 m × 0.25 mm × 0.25 μm, J&W Scientific Inc., Folsom, CA, USA) with a temperature program at 40 °C (3 min) to 230 °C (5 min) at 6 °C/min with helium as carrier gas (1.8 mL/min). The mass spectrometer was operated in electron impact mode (70 eV) and the masses were scanned over a range of 35–350 *m*/*z*. The transmission line temperature was 250 °C and the ion source temperature was 200 °C.

About 3 g wood fiber sample was added into a 15 mL headspace bottle and conditioned at 60 °C in a water bath for 40 min and the extraction occurred at the same temperature for 40 min. Desorption was carried out in the GC injection port.

Sensory assessments were carried out by a panel of four judges (two females and two males, 26 years old on average) from the Laboratory of Brewing Microbiology and Applied Enzymology at Jiangnan University. The panelists were trained for 3 months in GC-O using at least 30 odor-active reference compounds in a concentration 10 times above their odor thresholds in air. Sniffing time was approximately 40 min. During a GC run described above, the nose of a panelist was placed close to the sniffing port, responded to the aroma intensity of the stimulus, and recorded the aroma descriptor and intensity value as well as retention time. A six-point scale ranging from 0 to 5 was used for intensity judgment: 0 = none, 1 = very weak, 2 = weak, 3 = moderate, 4 = strong, and 5 = very strong.

The odorants were identified by comparing the MS spectra to the National Institute of Standards and Technology (NIST) library (https://www.nist.gov). The primary odor compounds were identified by mass spectrometry, retention time, and odor characterization.

## 4. Conclusions

About 40 kinds of odors and their relevant components of woods were identified by GC-O. These odorants were classified into alkanes, aldehydes, alcohols, aromatics, carboxylic acids, esters, and miscellaneous (silane derivatives). By comparing the time and intensity of odorants occurring in GC-O and that of VOCs occurring in GC-MS, no good relationship was found, which means that some high contents of VOCs did not contribute to the odors. The removal of extractives showed mostly reduced quantities and intensities of odors. However, some odors were unaffected. An increase of benzene residue was also observed. Many of the odors identified in woods were unpleasant to humans. The retention time was concentrated between 25 and 36 min described as stinky, burnt, leather, bug, herb, etc., which mainly originated from the thermos-oxidation of wood components. This study was helpful to understand the indoor air quality caused by wood. To mitigate the odor problem, a further study can focus on the effects of temperature on the odor release or try to find another effective odor elimination process.

## Figures and Tables

**Table 1:** Chemical components of woods.

Labels	Extractives (%) *	Holocelluloses (%)	α-Cellulose (%)	Lignin (%)
CP	2.87 (0.02)	61.33 (0.01)	44.24 (0.01)	23.26 (0.03)
RW	4.67 (0.03)	67.11 (0.02)	43.78 (0.02)	26.07 (0.01)

* The extractives refer to benzene-ethanol soluble extractives. The values in the parentheses are the deviations of four replicates.

**Table 2:** Identified key compounds from GC-MS of neat and extracted wood samples.

Peak Number	Retention Time (min)	Compounds	Classification	Percentage (%) *			
				CP	Extracted CP	RW	Extracted RW
1	2.92	Ethanol	Alcohol	2.3 (0.2)	1.4	1.9 (0.1)	1.0
2	4.23	Benzene	Aromatic	5.6 (0.6)	32.8 (0.5)	5.9 (0.2)	68.5 (0.9)
3	5.41	Acetic acid	Carboxylic acid	14.8 (0.2)	1.0	16.3 (0.2)	1.0
4	8.10	Hexanal	Aldehyde	6.6 (0.2)	-	1.2	0.3
5	11.13	Furfural	Aldehyde	1.0	-	1.5 (0.1)	0.3
6	14.50	Pentanoic acid	Carboxylic acid	1.8	-	0.4	0.3
7	15.06	1,3-Dichlorobenzene	Aromatic	1.6	-	2.1 (0.2)	0.5
8	18.12	Hexanoic acid	Carboxylic acid	10.8 (0.4)	0.8	1.9 (0.1)	0.3
9	18.94	Nonanal	Aldehyde	2.2 (0.2)	1.3	2.8 (0.2)	1.0
10	21.32	Heptanoic acid	Carboxylic acid	0.9	-	0.4	0.2
11	22.50	Tridecane	Alkane	3.5 (0.2)	3.0 (0.2)	3.2 (0.1)	1.5
12	24.46	Octanoic acid	Carboxylic acid	0.5	-	0.9	0.2
13	25.26	(*Z*)-2-Decenal	Aldehyde	2.8 (0.2)	2.1 (0.1)	2.2 (0.1)	1.6 (0.1)
14	25.73	Tetradecane	Alkane	9.2 (0.5)	9.0 (0.5)	8.3 (0.1)	3.2 (0.1)
15	26.21	Hexyltrimethoxysilane	Miscellaneous	0.2	-	-	-
16	26.53	4-Oxononanal	Aldehyde	0.2	-	-	-
17	26.78	(*E*)-2-Decenal	Aldehyde	0.7	-	-	-
18	27.51	Nonanoic acid	Carboxylic acid	0.9	0.4	2.3 (0.2)	0.6
19	28.78	Pentadecane	Alkane	3.0 (0.2)	4.0 (0.3)	3.5 (0.2)	1.3
20	29.02	Dodecanal	Aldehyde	2.2 (0.1)	1.6 (0.1)	1.4	0.6
21	29.27	*n*-Octyltriethoxysilane	Miscellaneous	1.5	-	1.2	0.2
22	30.79	(*E*)-6,10-Dimethyl-5,9-undecadien-2-one	Ketone	0.7	1.2	1.4	0.7
23	31.35	1-Dodecanol	Alcohol	3.6 (0.1)	3.1 (0.1)	6.8 (0.3)	3.0 (0.2)
24	31.69	Hexadecane	Alkane	1.3	2.0	1.9	0.7
25	32.98	2,6,10-Trimethyl-pentadecane	Alkane	0.5	1.0	1.0	0.4
26	34.05	2,4-di-*tert*-Butylphenol	Alcohol	1.0	0.7	3.4 (0.1)	0.4
27	34.25	2,6,10,14-Tetramethyl-pentadecane	Alkane	1.1	2.3	1.3	0.8
28	34.45	Tetradecanal	Aldehyde	-	-	0.7	0.4
29	34.76	Cedrol	Alcohol	0.6	1.2	1.0	0.6
30	35.40	2-Methyl-hetadecane	Alkane	-	-	0.5	-
31	35.90	(4-Octyldodecyl)-cyclopentane	Alkane	0.5	0.9	1.1	0.4
32	36.20	Benzoic acid 2-ethylhexyl ester	Ester	0.7	1.2	1.3	0.6
33	38.51	1,2-Benzenedicarboxylic acid bis(2-methylpropyl) ester	Ester	0.2	-	0.7	0.2
34	39.40	Dibutyl phthalate	Ester	-	-	0.6	0.5

* The percentage was calculated based on the peak area. The values in the parentheses are the deviations of four replicates. Deviations lower than 0.5% are not listed in the Table.

**Table 3:** Odorants and odor descriptors of neat and extracted wood samples.

Peak Number in GC-O/MS	Retention Time (min)	Odorant	Odor Descriptor	Intensity			
				CP	Extracted CP	RW	Extracted RW
1’/2	4.4	Benzene	Gasoline, solvent	1	3	1	5
2’/3	5.2	Acetic acid	Irritant, sour	2	2	2	2
3’/4	8.2	Hexanal	Bitter, green	3	0	0	0
4’/5	11.1	Furfural	Nut	3	0	3	0
5’/-	12.2	2-Furan-carboxaldehyde	Special, grain	2	0	0	0
6’/6	14.5	Pentanoic acid	Cheese, paint	4	4	4	4
7’/-	15.2	Octanal	Fruit, soap	5	0	0	0
8’/-	16.1	5-Methyl-2-furan-carboxaldehyde	Chocolate, coconut	0	0	4	4
9’/-	17.1	Decamethyl-cyclopentasiloxane	Bread, nut	3	0	4	4
10’/8	18.2	Hexanoic acid	Cheese, fishy	5	4	4	3
11’/-	18.7	2-Octenal	Bitter	0	0	3	0
12’/9	19.0	Nonanal	Flower, fat	4	3	4	3
13’/-	20.8	α-Methyl-α-2,5,7-octatrienyl-benzenemethanol	Bitter, nut	0	0	2	2
14’/10	21.2	Heptanoic acid	Stink, leather, bug	5	4	5	5
15’/-	21.9	2-Nonenal	Stink, fat, iris	5	5	4	4
16’/-	22.2	1-Methoxy-4-(2-propenyl)-benzene	Bitter, herb, burnt	5	5	4	4
17’/-	23.4	5,5,8-Trimethyl-3,6,7-nonatrien-2-one	Stink, chemical, bug	4	2	4	4
18’/12	24.4	Octanoic acid	Stink, fat, bug	5	4	5	5
19’/13	25.3	(*Z*)-2-Decenal	Stink, fat, herb	5	5	5	5
20’/15	26.1	Hexyltrimethoxysilane	Irritant, stink, herb	4	3	3	3
21’/-	26.3	Hexadecanoic acid	Fruit, sour	4	0	0	0
22’/16	26.5	4-Oxononanal	Herb	5	3	3	3
23’/17	27.0	(*E*)-2-Decenal	Kerosene, herb	4	4	4	4
24’/18	27.4	Nonanoic acid	Milk	4	1	4	2
25’/-	27.8	(*E*,*E*)-2,4-Decadienal	Animal, burnt	5	5	5	5
26’/-	27.9	3,7-Dimethyl-2,6-octadien-1-ol, acetate	Stink, bug	0	0	5	5
27’/-	28.4	2-Undecenal	Citrus, fishy, herb	0	0	5	3
28’/20	28.8	Dodecanal	Mint, pungent	4	4	4	4
29’/21	29.2	*n*-Octyl-triethoxysilane	Burnt, bug	5	3	5	3
30’/-	29.4	2-Methylpropanoic acid, 3-hydroxy-2,4,4-trimethylpentyl ester	Sour, bitter, herb	2	0	0	0
31’/-	30.3	Decanoic acid	Herb	2	0	4	0
32’/-	31.0	8-Methyl-1-undecene	Paint, dirt	5	5	5	5
33’/-	31.7	1-Tetradecanol	Dirt, bronze	5	0	0	0
34’/-	32.1	1-Pentadecanol	Bronze	4	4	0	0
35’/25	32.8	2,6,10-Trimethyl-pentadecane	Gasoline, bronze	3	3	4	3
36’/29	34.6	Cedrol	Fat, wood	3	3	3	3
37’/-	35.9	Octadecanal	Bronze, grease	4	0	4	2
38’/32	36.3	Benzoic acid, 2-ethylhexyl ester	Milk, fruit	4	4	4	4
39’/33	38.8	1,2-Benzene dicarboxylic acid, bis(2-methylpropyl) ester	Cake, grass	3	0	5	3

The unpleasant odors are highlighted in character shading. The intensities were determined on agreements of four judges.

**Table 4:** Average growth ring widths, densities, and origins of place of the woods.

Label	Wood	Scientific Name	Average Growth Ring Width (cm)	Density (g/cm^3^) *	Origin of Place
CP	Cathy poplar	*Populus cathayana* Rehd.	0.6	0.49–0.52	Hebei, China
RW	Rubberwood	*Hevea brasiliensis*	0.2	0.64–0.67	Hainan, China

* The density values of six replicates were tested at moisture contents about 12%.
